# The Versatile Applications of Antisense Oligonucleotides in Modern Medicine

**DOI:** 10.3390/ijms27125612

**Published:** 2026-06-22

**Authors:** Xue-Hai Liang, Lingdi Zhang

**Affiliations:** Arnatar Therapeutics Inc., 4930 Directors Place, Suite 120, San Diego, CA 92121, USA; ldzhang@arnatar.com

**Keywords:** antisense oligonucleotide, ASO, regulation, degradation, modification, translation, splicing

## Abstract

Antisense oligonucleotides (ASOs) are a class of nucleic acid therapeutics that modulate gene expression through diverse mechanisms. Since their initial demonstration in inhibiting viral genes, advances in medicinal chemistry, pharmacology, and delivery have enabled robust and durable target engagement across multiple tissues. Chemical modifications to the backbone, ribose, and nucleobases have improved nuclease resistance, binding affinity, and pharmacokinetics, while conjugation and delivery technologies have expanded tissue accessibility. Beyond classical RNase H–mediated RNA degradation, ASOs regulate gene expression via splicing modulation, microRNA inhibition, transcriptional activation, and translation modulation, supporting both gene silencing and upregulation strategies. Multiple ASO drugs are now approved, particularly for genetic diseases, with many more in clinical development. This review outlines the evolution of antisense technology, key chemical and delivery innovations, ASO pharmacokinetics and intracellular trafficking, the mechanisms underlying gene regulation, and current clinical applications and future opportunities.

## 1. Introduction

Antisense oligonucleotides (ASOs) are short synthetic nucleic acids designed to bind complementary RNA sequences and modulate gene expression through sequence-specific hybridization. The antisense concept was first demonstrated in 1978 when Zamecnik and Stephenson showed that synthetic oligodeoxynucleotides could inhibit replication of Rous sarcoma virus by hybridizing to viral RNA [1]. This seminal observation established the principle that nucleic acid sequences could be used as programmable drugs capable of targeting specific RNA molecules.

Unlike traditional small-molecule drugs, which typically interact with proteins, ASOs rely primarily on Watson–Crick base pairing to recognize their target RNAs. This feature enables a rational design model in which the therapeutic agent can be directly designed based on genomic sequence information. The concept of antisense therapy, therefore, represents a paradigm shift in drug discovery, allowing precise targeting of disease-associated genes that may be difficult to address using conventional pharmacological approaches.

Early enthusiasm for antisense therapeutics was tempered by several technical challenges. Native oligonucleotides composed of phosphodiester (PO) linkages are rapidly degraded by nucleases in biological fluids, resulting in poor stability and limited pharmacological activity [2]. In addition, early antisense molecules exhibited inefficient cellular uptake and limited distribution to target tissues [3]. Safety concerns, including immune activation and off-target effects, also posed obstacles to clinical development [4].

Over the past four decades, intensive efforts in medicinal chemistry, pharmacology, and molecular biology have addressed these challenges. A series of chemical modifications to the oligonucleotide backbone, sugar moiety, and nucleobases have dramatically improved stability and pharmacokinetic properties [5]. Advances in delivery technologies and conjugation strategies have further enhanced tissue targeting and cellular uptake. Biological research and genomic sequencing have enabled identification of new targets and new mechanisms of gene expression regulation using ASOs. Modulation of gene expression using ASOs can occur at different steps, such as transcription, pre-mRNA processing including splicing and alternative polyadenylation, mRNA translation, and mRNA degradation. Combined with biological mechanisms, ASOs can be designed to down-regulate or up-regulate gene expression. These developments have transformed antisense oligonucleotides from research tools into clinically viable therapeutics.

Today, antisense technology represents one of the most advanced nucleic acid therapeutic platforms [6]. Recent reviews have further highlighted the growing impact of ASO therapeutics in monogenic diseases and the continued expansion of oligonucleotide medicines into broader therapeutic areas, supported by advances in chemistry, delivery technologies, and target identification [7]. More than a dozen ASO drugs have received regulatory approval worldwide [8,9,10], targeting diseases including spinal muscular atrophy (SMA), familial hypercholesterolemia, transthyretin amyloidosis, Duchenne muscular dystrophy (DMD) and amyotrophic lateral sclerosis (ALS). Numerous additional candidates are currently being evaluated in clinical trials for a wide range of diseases.

In this review, we examine the major technological advances that have enabled the maturation of antisense therapeutics. We discuss chemical modifications that enhance ASO pharmacological performance, delivery strategies that facilitate tissue targeting, and emerging insights into ASO intracellular trafficking. We also highlight the diverse mechanisms by which antisense oligonucleotides modulate gene expression and summarize current therapeutic applications and future opportunities for this rapidly evolving class of medicines.

## 2. Chemical Modifications Enhancing ASO Pharmacological Performance

Native oligonucleotides are rapidly degraded in vivo and exhibit limited pharmacological activity. Chemical modifications of nucleic acids have therefore been developed to improve nuclease resistance, increase binding affinity to RNA targets, enhance pharmacological properties, and reduce toxicity.

### 2.1. Backbone Modifications

Backbone modification represents one of the most critical advances in the development of ASO therapeutics, as it helps prevent the rapid degradation of native oligonucleotides due to phosphodiester (PO) linkages and improves ASO pharmacokinetic properties. Chemical modification of the phosphate backbone, therefore, plays a central role in improving metabolic stability, tissue distribution, and overall pharmacological performance of ASOs [2,6].

**Figure 1:**
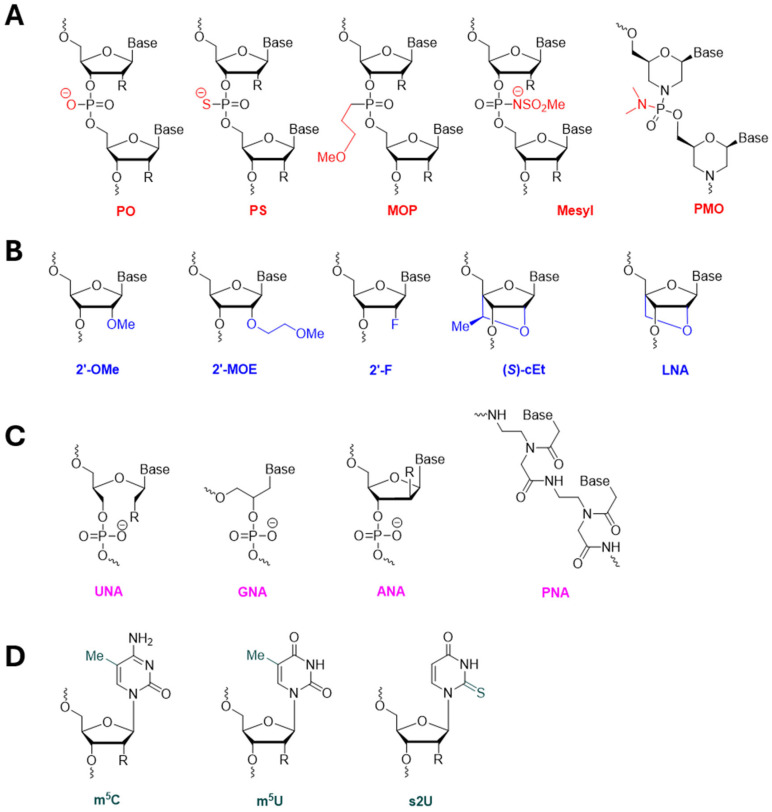
Commonly used chemical modifications of ASOs. (**A**) Backbone modifications. PO, phosphodiester; PS, phosphorothioate; MOP, methoxypropyl phosphonate; Mesyl, methanesulfonyl; PMO, phosphorodiamidate. (**B**) 2′ modifications. Me, 2′-O-methyl; MOE, methoxyethyl; F, fluoro. cEt, constrained ethyl; LNA, locked nucleic acid; (**C**,**D**) Base modifications. m5C, 5-Methylcytosine; m5U, 5-Methyluridine; s2U, 2-Thiouridine.

The most widely used backbone modification is the phosphorothioate (PS) linkage, in which one of the non-bridging oxygen of the phosphate group is replaced by sulfur (Figure 1A). This substitution substantially increases resistance to nucleases, dramatically prolonging the stability of oligonucleotides in serum and intracellular environments [11,12]. In addition, the sulfur substitution increases hydrophobicity and promotes binding to plasma proteins [13]. This protein binding reduces rapid renal filtration and prolongs circulation time, allowing ASOs to distribute efficiently to tissues following systemic administration [14].

PS linkages also facilitate cellular uptake. PS-modified ASOs interact with various cell-surface proteins, including scavenger receptors and other membrane-associated proteins [3,15]. These interactions enable efficient uptake of ASOs without the need for complex delivery systems, a property that distinguishes ASO therapeutics from other nucleic acid drugs such as siRNAs. However, the substitution of sulfur introduces a chiral center at each phosphate linkage, resulting in mixtures of stereoisomers across the oligonucleotide backbone. These stereochemical differences can influence interactions with proteins, stability, and pharmacological activity of ASOs [16]. For example, it has been shown that R-form PS can enhance RNase H1 cleavage, whereas S-form PS can enhance protein binding property [16,17,18]. The effects of chiral PS are often position and context dependent [12,16,19,20].

Although PS has become the dominant backbone modification in antisense therapeutics, alternative backbone structures have also been developed to improve pharmacological properties. One example is the phosphorodiamidate morpholino oligomer (PMO), in which the ribose sugar is replaced by a morpholine ring, and the phosphodiester linkage is replaced with a phosphorodiamidate bond (Figure 1A). This backbone is electrically neutral, which reduces interactions with proteins and contributes to metabolic stability [21]. PMOs are resistant to nuclease degradation and have demonstrated favorable safety profiles in clinical applications, particularly in splicing-modulation therapies for Duchenne muscular dystrophy [22,23]. However, the neutral backbone also reduces interactions with proteins in cellular uptake pathways, often requiring higher doses and/or specialized delivery approaches [24].

Additional backbone chemistries continue to be explored. Methanesulfonyl (Mesyl) phosphoramidate linkages (Figure 1A), for example, introduce modified phosphoramidate groups that alter backbone polarity and may influence nuclease stability and RNA binding properties, improving ASO drug performance [25,26,27]. These modifications provide alternative approaches to tuning ASO pharmacology, potentially improving therapeutic performance or enabling new mechanisms of action. In addition, other backbone modifications, such as methoxypropylphosphonate (MOP) and peptide nucleic acids (PNA), were also evaluated and demonstrated certain advantages [28,29]; however, the performance in clinics is yet to be further determined.

### 2.2. Ribose Modifications

Modifications at the 2′ position of the ribose represent a major advance in ASO chemistry (Table 1). These modifications profoundly influence the physicochemical and pharmacological properties of ASOs by increasing resistance to nuclease degradation, enhancing RNA binding affinity, and reducing activation of innate immune responses. Substitutions at the 2′ position also influence ribose conformational preference, typically stabilizing the C3′-endo sugar pucker, which favors formation of an A-form duplex when hybridized to RNA [2,30].

Although multiple modifications have been evaluated, only several 2′ ribose modifications are widely used in ASO therapeutics, including 2′-O-methyl (2′OMe), 2′-O-methoxyethyl (2′MOE), constrained ethyl (cEt), locked nucleic acid (LNA), and 2′-Fluoro (2′-F) (Figure 1B). These modifications differ in steric bulk, conformational constraint, hydrophobicity, and affinity for RNA, which in turn influence potency, pharmacokinetics, and safety profiles.

The 2′OMe modification improves nuclease resistance. This modification modestly increases RNA binding affinity and has been widely used in siRNA and early ASO applications [2]. In addition, 2′OMe reduces activation of innate immune receptors such as TLR7 and TLR8, improving tolerability in vivo [31]. Similarly, the 2′MOE modification introduces a larger methoxyethyl substituent at the 2′ position. This group strongly favors the C3′-endo conformation and enhances hybridization affinity and nuclease resistance, and also improves ASO safety profile relative to 2′OMe [32]. As a result, MOE chemistry forms the basis of many clinically approved ASO drugs, including mipomersen, inotersen, nusinersen, and volanesorsen [14].

LNA represents one of the most powerful affinity-enhancing modifications. In LNA nucleotides, a methylene bridge connects the 2′ oxygen and the 4′ carbon of the ribose, rigidly locking the sugar into the C3′-endo conformation. This structural constraint dramatically increases the stability of ASO/RNA duplex. However, the high RNA binding affinity and strong protein interactions of LNA modified ASOs may raise safety concerns if used excessively, necessitating careful design [2,33].

Similar to LNA, the cEt modification also locks the ribose into the C3′-endo conformation. This significantly increases RNA binding affinity relative to MOE. As a result, cEt-modified ASOs often display improved potency in vivo, enabling shorter ASO lengths or lower therapeutic doses [34]. In addition, cEt modification also enhances protein binding compared with MOE modifications [35,36]; thus, a careful balance of RNA binding affinity and protein binding affinity needs to be considered.

A comparison of these modifications is shown in Table 1. These 2′ modifications are commonly incorporated into gapmer ASOs, in which a central DNA region capable of recruiting RNase H is flanked by modified nucleotides. For splicing modulation ASOs, commonly used are PS/MOE ASOs or PMO ASOs without DNA, to avoid target RNA degradation.

### 2.3. Alternative Nucleic Acid Analogs

Another class of sugar modifications includes arabinonucleic acid (ANA) and related analogs, as well as backbone–sugar hybrid modifications such as glycol nucleic acid (GNA) or unlocked nucleic acid (UNA) [37,38,39] (Figure 1C). ANA nucleotides alter the orientation of the 2′ substituent relative to RNA, which can modulate duplex geometry and reduce off-target interactions while maintaining gene-silencing efficiency [37,40]. Similarly, GNA and UNA insertions that reduce RNA binding affinity can modulate duplex flexibility and thermodynamic asymmetry of siRNA duplexes, thereby improving strand selection and specificity during RNA-induced silencing complex (RISC) loading when placed at certain positions of the antisense strand of siRNAs [38,41].

### 2.4. Base Modifications

In addition to backbone and ribose modifications, nucleobase modifications are used to optimize ASO performance. Base modifications can influence RNA binding affinity, mismatch discrimination, and immune recognition, while generally preserving the overall geometry of the duplex [42]. Compared with backbone or sugar modifications, base modifications typically produce more modest structural changes but can provide useful improvements in potency and safety when incorporated strategically [2,19].

One commonly used modification is 5-methylcytosine (m^5^C) (Figure 1D), which enhances base stacking interactions and typically increases duplex melting temperature (Tm) by approximately 0.5–1 °C per modification [43], thereby improving RNA binding affinity. In addition, m^5^C has been reported to reduce immune stimulation and improve pharmacological stability, and it is incorporated into many therapeutic ASO designs to reduce the immunoreactivity of CpG sequences [14,44]. Other nucleobase analogs, such as pseudouridine and modified uridines (m^5^U, s2U), can stabilize RNA duplexes and may reduce immune activation. More strongly affinity-enhancing modifications, including 5-propynyl-modified pyrimidines, significantly increase duplex stability. However, excessive stabilization may increase the risk of off-target hybridization [45].

## 3. Mechanisms of Antisense-Mediated Gene Down-Regulation

**Figure 2:**
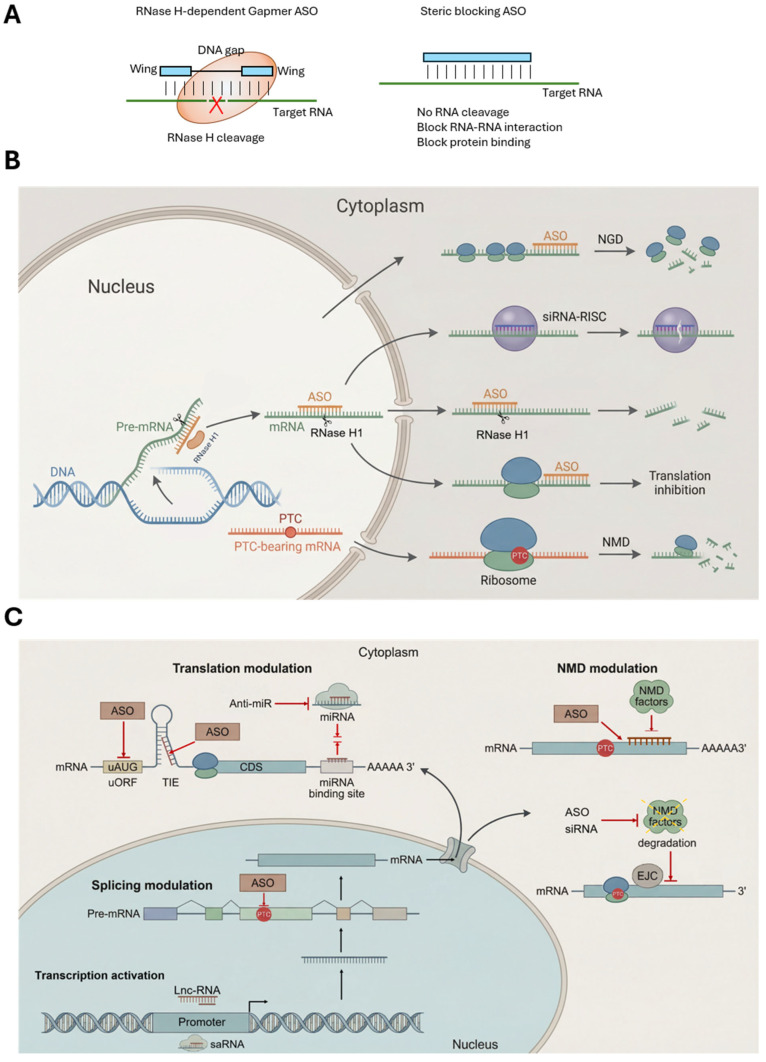
Antisense technology can modulate gene expression through different mechanisms. (**A**) Mechanisms of ASO-mediated gene expression regulation. Left panel, RNase H-dependent, gapmer ASO-mediated enzymatic cleavage of target RNA. Right panel: steric-blocking ASOs that may modulate RNA or protein binding to the target RNA. (**B**) Common antisense mechanisms for downregulation of gene expression include RNase H-dependent ASOs, which function in both the nucleus and cytoplasm; and steric-blocking ASOs, which modulate splicing to induce nonsense-mediated decay (NMD) via inclusion of premature termination codons (PTCs) or inhibit translation. (**C**) Antisense approaches can also upregulate gene expression through multiple mechanisms. Nuclear mechanisms include transcriptional activation (e.g., via small activating RNAs, saRNAs) and targeting of long noncoding RNAs, as well as splicing modulation to prevent formation of PTC-containing transcripts. In the cytoplasm, ASOs may enhance translation efficiency by suppressing translation-inhibitory elements, or increase mRNA levels by inhibiting NMD-mediated decay. NGD, no-go decay; NMD, nonsense-mediated decay; EJC, exon–exon junction complex; uORF, upstream open reading frame; TIE, translation inhibitory element.

Once released from endosomes/lysosomes, ASOs distribute in the cytoplasm and the nucleus, where ASOs can act through different mechanisms that either down-regulate or up-regulate gene expression, depending on the chemistry and binding position in target RNAs (Figure 2A). For down-regulation, the most common mechanism is RNase H–mediated degradation. ASOs can also suppress protein production through steric-blocking mechanisms, such as preventing translation or altering splicing to induce nonsense-mediated decay (NMD) (Figure 2B). Conversely, ASOs can increase gene expression by modulating RNA transcription, processing or stability, and translation (Figure 2C). Through these diverse mechanisms, ASOs provide a versatile platform for precisely modulating gene expression in both directions, enabling therapeutic strategies for diseases caused by either gain-of-function or loss-of-function alterations [6,14,30].

### 3.1. RNase H–Mediated RNA Degradation

RNase H-dependent gapmer ASOs represent the most widely used design for therapeutic gene knockdown (Figure 2A). Gapmers consist of a central “gap” region of DNA nucleotides flanked by 2′ modified nucleotides (often 2′-MOE, cEt, or LNA) that improve stability and binding affinity. When the gapmer hybridizes to complementary RNA, the DNA–RNA heteroduplex is recognized by RNase H1, an endonuclease that selectively cleaves the RNA strand in the duplex. The flanking modified nucleotides protect the ASO from nuclease degradation and increase hybridization affinity, while the central DNA region, typically 8–10 nucleotides in length, is required for RNase H recruitment and enzymatic activity [6,14].

Gapmer ASOs offer several advantages for therapeutic development. The catalytic RNase H mechanism enables potent and sustained knockdown at relatively low intracellular concentrations, and because RNase H1 is present in both the cytosol and nucleus, gapmer ASOs can degrade RNAs in both cellular compartments [46,47]. However, high-affinity binding to RNA and strong protein interactions may increase the risk of off-target hybridization or nonspecific protein binding, leading to sequence/chemistry-dependent toxicity. Thus, optimization may sometimes be required, for example, by altering chemical modifications [48,49,50,51]. As a comparison, siRNAs act through the RNA interference (RNAi) pathway, where the cleavage of target mRNA occurs mainly in the cytoplasm, although RNAi-mediated knockdown has also been reported for nuclear RNAs [52,53]. RNAi-mediated cleavage is highly efficient and can produce strong and durable knockdown. As a result, siRNA therapeutics have been particularly successful for liver targets through GalNAc conjugation. On the other hand, ASOs offer broader versatility for extrahepatic tissues, nuclear targets, and splicing modulation applications, making the two platforms complementary in therapeutic development.

### 3.2. Translational Inhibition

ASOs can be designed as steric blocking compounds (Figure 2A,B). ASOs can inhibit protein production through translation-blocking mechanisms, in which the ASO binds to specific regions of the mRNA, such as the 5′ UTR and start codon, and sterically prevents ribosome assembly or elongation [54]. However, persistent ribosome stalling caused by ASO binding can trigger the no-go decay (NGD) pathway [55], a surveillance mechanism that recognizes stalled ribosomes and promotes endonucleolytic cleavage and degradation of the affected mRNA. Translation-blocking ASOs are typically fully modified without DNA nucleotides, thus do not recruit RNase H1. Compared with RNase H1–dependent gapmer ASOs, translation-blocking ASOs can offer greater specificity for regulatory regions and avoid enzymatic cleavage that may generate unintended RNA fragments. However, higher intracellular occupancy is generally required because the activity is not catalytic, which may limit potency and durability relative to RNase H–mediated mechanisms [3,14].

### 3.3. Splicing Modulation to Induce Non-Sense Mediated Decay (NMD)

Pre-mRNA splicing was first recognized in 1977, a process that removes the introns and reconnects neighboring exons into mature mRNAs [56,57]. Subsequent work demonstrated that many genes undergo alternative splicing, a process in which different combinations of exons are joined to generate multiple mRNA isoforms from a single gene, greatly expanding proteomic diversity [58]. Alternative splicing is affected mainly by splicing factors that bind to certain positions of pre-mRNA to modulate splice site utilization. Taking advantage of these observations, ASOs can modulate this process by binding to splice sites or regulatory elements within pre-mRNA, thereby altering spliceosome recognition by preventing the binding of splicing factors. As a result, ASOs can promote exon skipping or exon inclusion, enabling the formation of a different splicing isoform that may cause an open-reading frame shift to form a premature stop codon (PTC). The presence of PTC can reduce mRNA levels by triggering non-sense-mediated decay (NMD) [59]. Splicing modulation ASOs are typically fully modified, e.g., using 2′OMe, MOE, PMO, or LNA chemistries, which provide high RNA binding affinity and nuclease resistance but do not support RNase H activity. This approach is useful when RNase H-dependent ASO is not favorable, for example, due to off-target cleavage risks or safety concerns. Such ASOs are generally 18–25 nucleotides in length to achieve strong hybridization and specificity [60].

### 3.4. MicroRNA Mimics

MicroRNA mimics provide an alternative strategy to reduce protein expression by restoring or enhancing endogenous miRNA regulatory pathways. miRNAs are ~22-nt noncoding RNAs that partially base-pair with complementary sequences, typically in the 3′UTR of target mRNAs, resulting in translational repression and accelerated mRNA decay [61]. Since miRNAs modulate gene expression through the seed region that can form 7–8 base-pairing with target mRNAs, not surprisingly, each miRNA may modulate hundreds of mRNA substrates. Therapeutic miRNA mimics are synthetic double-stranded RNAs designed to replenish disease-relevant miRNAs. After delivery into cells, the guide strand is incorporated into RISC and suppresses multiple transcripts sharing the same seed sequence. This approach has been evaluated clinically, for example, MRX34, a liposomal miR-34a mimic tested in phase I oncology trials that showed target engagement but was discontinued due to immune-related toxicities [62]. Compared with RNase H–mediated ASOs, miRNA mimics can simultaneously reduce the expression of the entire gene networks of the miRNA targets, which may be advantageous for complex diseases, but this broader activity also increases the risk of off-target effects [61,63].

## 4. Mechanisms of ASO-Mediated Gene Expression Up-Regulation

Many diseases are caused by insufficient levels of functional proteins; thus, increasing the levels of such proteins will be an attractive approach to treat the disease from the root cause [64]. Although different approaches have been developed, such as mRNA delivery, gene therapy, and protein replacement, these methods require specialized delivery of macromolecules and are often accompanied by safety challenges or difficulty in controlling protein levels [65]. Increasing endogenous proteins to an effective and safe level using smaller compounds with mature platforms provides a more desired approach. It has been shown that ASOs can upregulate gene expression through different mechanisms that enhance mRNA production, stability, or translation (Figure 2C).

### 4.1. Transcription Activation

Antisense oligonucleotide-mediated transcriptional activation has been explored through several approaches that modulate gene expression regulation at the chromatin or promoter level. One strategy involves targeting promoter-associated RNAs or promoter regions using short duplex RNAs known as small activating RNAs (saRNAs), which recruit Argonaute-containing complexes and transcriptional cofactors to promoter sequences, resulting in increased RNA polymerase II recruitment and transcriptional activation [66,67]. This mechanism, termed RNA activation (RNAa), likely involves DNA methylation and regulation of nucleosome structure [68]. Several mRNAs, including p21 and E-cadherin [69,70], have been shown to be increased using this approach, and certain saRNA targets have advanced into clinical testing. For example, MTL-CEBPA, an saRNA therapeutic designed to activate the transcription factor CEBPA, has been evaluated in patients with hepatocellular carcinoma and showed evidence of pharmacodynamic target activation and acceptable safety in early clinical trials (NCT02716012) [66,71].

A second approach involves ASO-mediated inhibition of repressive long noncoding RNAs (LncRNAs) or natural antisense transcripts (NATs) that suppress transcription of target genes [72]. These antisense transcripts can interfere with transcription elongation, recruit chromatin-modifying complexes, or promote repressive epigenetic states [73]. By selectively degrading such LncRNAs, ASOs can relieve transcriptional repression and restore expression of the corresponding gene. A well-known example is BDNF-AS, where ASO-mediated knockdown of the antisense lncRNA increased BDNF transcription in rodent models [74]. Similarly, ASOs targeting UBE3A-ATS can unsilence the paternal *UBE3A* allele in neurons, an approach currently explored clinically for Angelman syndrome [75].

A related concept is the RegRNA (regulatory RNA) approach, in which ASOs target regulatory RNA elements within gene loci that control transcriptional output [76]. These regulatory RNAs can include promoter-associated transcripts, enhancer RNAs, or antisense RNAs that influence chromatin architecture and transcription factor recruitment. By modulating these regulatory RNA species, ASOs can indirectly alter the transcriptional activity of the associated gene. Although still largely in preclinical development, studies in rodent models have demonstrated that targeting regulatory RNAs can increase expression of genes involved in metabolic and neurological pathways [77].

Despite promising early results, transcription-activating ASO approaches remain less mature than gene-silencing strategies. Key uncertainties include the mechanistic complexity of promoter and chromatin regulation, variability in transcriptional activation across cell types, and the durability of gene upregulation. In addition, relatively low conservation in LncRNA sequence and regulation can complicate translation from in vitro to in vivo, and from rodents to humans. Nevertheless, continued advances in understanding regulatory RNA biology and chromatin dynamics are expected to expand the therapeutic potential of ASO-mediated gene expression activation.

### 4.2. ASO-Mediated Splicing Modulation to Increase Protein Levels

ASOs can increase protein expression by modulating pre-mRNA splicing to restore or enhance the production of functional transcripts. These splicing modulation ASOs bind to specific sequences in pre-mRNA, such as splice sites or regulatory motifs including exonic or intronic splicing silencers (ESS/ISS) and enhancers (ESE/ISE). By blocking the binding of splicing factors, ASOs redirect spliceosome recognition to promote exon inclusion or exclusion, preventing production of PTC-containing mRNA, thereby restoring the correct reading frame and increasing the amount of productive mRNA. This strategy is particularly useful for diseases caused by haploinsufficiency or aberrant splicing, where increasing the proportion of correctly spliced transcripts leads to increased protein expression [6,78].

One of the most prominent examples is nusinersen, an ASO therapy for SMA [79]. Nusinersen binds to an intronic splicing silencer in the *SMN2* gene, promoting inclusion of exon 7 and restoring production of full-length SMN protein in motor neurons. In addition, PMO ASOs have also been used to alter splicing and restore partial function of dystrophin to DMD [80]. Similar strategies are being explored for other genetic diseases, including *SCN1A* haploinsufficiency in Dravet syndrome [81], *OPA1*-associated optic neuropathy [82], and certain metabolic disorders.

Splicing modulation ASOs offer several advantages. They allow precise regulation of endogenous gene expression without altering genomic DNA, and because the mechanism is steric blocking rather than enzymatic degradation, they can be designed with fully modified chemistries such as PMO or 2′-modified ASOs that provide high stability and reduced off-target cleavage. However, the approach also has challenges. Since the ORF must be maintained after splicing alternation and the altered protein needs to remain functional, limited targets can be modulated with this approach. In addition, splicing regulation is often cell-type and context dependent, requiring careful identification of effective regulatory sites, and the magnitude of protein increase may vary depending on the baseline splicing pattern of the target gene. Furthermore, efficient delivery to certain tissues, such as skeletal muscle or brain, remains a challenge and may require specialized delivery strategies. Nevertheless, ASO-mediated splicing modulation has become one of the clinically validated oligonucleotide therapeutic mechanisms [8,78].

### 4.3. ASO-Mediated mRNA Stabilization

ASOs can increase protein expression by stabilizing mRNA transcripts, thereby prolonging transcript lifetime and enhancing translation. One well-established mechanism involves anti-miRNA ASOs (antagomirs), which bind to and inhibit microRNAs that normally repress gene expression. By blocking specific microRNAs, anti-miRNA ASOs prevent this repression and increase expression of the miRNA-regulated genes. This approach has advanced into clinical development. For example, anti-miR-122 ASOs have been tested for treatment of hepatitis C virus infection and metabolic diseases, demonstrating effective inhibition of miRNA activity and increased expression of miRNA-regulated genes [83,84]. Another example is anti-miR-17 ASO (Farabursen, RGLS8429) developed to treat autosomal dominant polycystic kidney disease (ADPKD). MiR-17 suppresses expression of *PKD1* and *PKD2*, which encode the polycystin proteins that regulate kidney cell growth. Inhibition of miR-17 increases polycystin levels and may slow cyst growth in ADPKD models and in patients [85,86]. In addition, PNA ASOs have been designed to target pre-miRNA junctions to inhibit miRNA maturation, thereby increasing gene expression of miRNA targets [87].

ASOs can also stabilize mRNA by directly blocking regulatory elements within the transcript. For instance, ASOs can bind to miRNA binding sites within the 3′UTR to prevent miRNA binding [88], a strategy sometimes referred to as target site blockers. In addition, ASOs can block binding sites for RNA-binding proteins that promote mRNA degradation [89], thereby protecting transcripts from decay pathways. These steric-blocking mechanisms can selectively increase expression of specific transcripts while preserving normal regulatory networks. mRNA-stabilizing ASOs offer an approach to enhance endogenous protein production, particularly for diseases caused by reduced gene expression. However, the magnitude of upregulation may vary depending on cellular context and competing regulatory mechanisms [6,90].

### 4.4. ASO-Mediated Translation Enhancement

ASOs can enhance protein expression by increasing translation efficiency of existing mRNA transcripts, typically through steric blocking of inhibitory elements located in UTRs. Many mRNAs contain regulatory features in the 5′UTR or 3′UTR that suppress translation, including upstream open reading frames (uORFs), translation inhibitory elements (TIEs), RNA structures such as G-quadruplexes, and binding sites for translational repressors [91,92]. By binding to these regions, ASOs can prevent inhibitory RNA structures from forming or block the access of regulatory proteins that interfere with ribosome function, thus lifting the suppression of these elements and enhancing translation efficiency. To avoid RNA degradation, translation-enhancing ASOs are generally designed as fully modified blocking ASOs [6,93,94,95].

One mechanism involves ASOs targeting uORFs [93,96]. uORFs are common translational regulatory elements in 5′UTRs that divert ribosomes away from the primary coding sequence [97]. Blocking the uORF start codon or surrounding regulatory sequences with an ASO allows ribosomes to bypass the translation of uORF and enhance translation at the primary open reading frame (pORF) [93]. This strategy has been demonstrated in several disease models involving haploinsufficiency [98,99], where moderate increases in protein expression can restore physiological function. For example, ASOs targeting uORF in Jagged1 mRNA have been shown to increase protein expression and improve disease phenotypes in mouse models of Alagille syndrome [100], illustrating the therapeutic potential of translation-enhancing ASOs.

Other translational regulatory elements can also be targeted by ASOs. TIEs and G-quadruplex structures within the 5′UTR can impede ribosome scanning or initiation. ASO binding can destabilize these structures and restore more efficient translation [94,95]. Similarly, ASOs can block binding sites for RNA-binding proteins that suppress translation, thereby increasing protein levels. These steric-blocking approaches enable selective enhancement of endogenous protein production [78,101].

More recently, new RNA-based approaches have been explored to improve translation efficiency by enhancing recruitment of translation-related proteins to target mRNAs. Translation-activating RNAs (taRNAs) recruit translation initiation proteins eIF3 and eIF4G through an artificial internal ribosome entry site (IRES) provided by the exogenous RNA, which also base-pairs with the target mRNA [102]. Another approach, the tethered mRNA amplifier, co-expresses a guide RNA to bind target mRNA and recruits dCas13-PABP fusion protein to increase mRNA and protein levels [103]. Due to the sizes and chemical properties of the above-mentioned RNAs, facilitated delivery is required, such as through LNPs. On the other hand, we recently developed a new upregulation technology, termed antisense-coupled translation upregulation (ACT-UP1), which enhances translation efficiency of target mRNAs without increasing mRNA levels. ACT-UP1 employs drug-like, fully modified ASOs without the need for facilitated delivery. ACT-UP1 ASOs contain an antisense segment to specifically base-pair with the target mRNA, and also contain a short segment that does not base-pair with the mRNA but is designed to recruit endogenous translation-enhancing protein(s) to increase translation efficiency in vitro and in vivo [104]. Since these approaches do not need to target inhibitory elements in mRNAs, the number of suitable targets is dramatically increased. Although many translation-enhancing ASO strategies remain in preclinical or early translational stages, they represent a promising avenue for treating diseases caused by reduced gene expression, particularly in tissues where delivery of replacement proteins or gene therapy may be challenging.

## 5. Delivery Strategies for ASOs

ASO drugs act on cellular RNAs, and thus need to be delivered into cells and proper cellular compartments. Delivery is a central challenge for nucleic acid therapeutics because these molecules are large, polyanionic, and generally unable to cross cellular membranes efficiently. However, ASOs possess properties that distinguish them from other nucleic acid drugs such as siRNAs, allowing broader tissue distribution without complex delivery systems [2,6].

Single-stranded ASOs, particularly those containing PS backbones, bind plasma and cell surface proteins that facilitate adsorptive and receptor-mediated endocytosis [3,13,35,105]. Through these interactions, ASOs can enter cells directly following systemic administration [3]. This uptake mechanism enables efficient accumulation in highly endocytic tissues such as the liver and kidney [14]. Through intrathecal injection, PS modified ASOs can also enter the CNS and act in neuronal cells, such as splicing modulating ASO nusinersen for SMA, and RNase H-dependent gapmer ASO Tofersen for ALS [106]. Similarly, ASOs can be delivered locally, such as to the eye through intravitreal injection [107]. Indeed, a splicing modulating ASO STK-002 targeting *OPA1* is currently in a clinical trial to treat Autosomal Dominant Optic Atrophy [108]. These physicochemical properties of PS-modified ASOs enable pharmacodynamics in different tissues, albeit with different efficiency that could be improved through targeted delivery.

In contrast, siRNAs are double-stranded molecules that exhibit limited protein interaction and poor intrinsic cellular uptake. Similarly, PMO ASOs also have poor protein-binding affinity. As a result, these compounds typically require delivery systems such as lipid nanoparticles (LNPs) or ligand conjugates. These differences in uptake mechanisms represent a key distinction between ASO and siRNA therapeutics.

### 5.1. Lipid Nanoparticles

LNPs have become one of the most successful delivery platforms for nucleic acid therapeutics, particularly for siRNA and mRNA. Although LNP delivery for ASOs is less common because of the high protein-binding capacity and efficient cellular uptake of ASOs, LNP-mediated ASO therapeutics have been explored, such as prexigebersen and BP1001. The development of modern LNP systems evolved from early liposome formulations and was enabled by the discovery of ionizable lipids, which remain largely neutral at physiological pH but become positively charged in acidic endosomes [109], thereby promoting endosomal escape of nucleic acids. This platform achieved clinical validation with the approval of the siRNA drug patisiran and was further accelerated by the large-scale use of LNPs in mRNA vaccines for COVID-19. Typical LNP formulations contain four key components: ionizable lipids, helper phospholipids, cholesterol, and PEG-lipids. Each component contributes to particle stability, encapsulation efficiency, and intracellular delivery [110]. LNPs effectively protect nucleic acids from degradation and enhance cellular uptake. However, Challenges of application of LNPs exist, including preferential accumulation in the liver, incomplete endosomal escape, potential risks of immune activation, and manufacturing complexity [111].

To expand the range of tissues accessible to nucleic acid therapeutics, significant efforts have focused on customizing LNP composition and targeting strategies. Modifying ionizable lipids or incorporating selective organ targeting (SORT) lipids can shift biodistribution toward organs such as the lung or spleen in preclinical models [112]. Additional strategies include conjugation or decoration of LNPs with targeting ligands, peptides, or proteins to promote uptake of LNPs by specific cell types, as well as adjusting lipid composition to improve endosomal escape and intracellular trafficking [113]. Despite these advances, efficient systemic delivery to tissues beyond the liver, such as muscle, the central nervous system, and tumors, remains challenging. Future directions in the LNP field, therefore, focus on developing next-generation ionizable lipids with improved endosomal escape, organ-selectivity, biodegradable lipid structures to improve safety, and scalable manufacturing technologies that enable consistent product quality [114,115,116].

### 5.2. Ligand-Conjugated Delivery

Ligand-mediated delivery has become an important strategy to enhance the tissue and cell-type specificity of oligonucleotide therapeutics. By conjugating targeting ligands to ASOs or siRNAs, uptake can be directed through receptor-mediated endocytosis in specific cell populations, improving potency and reducing systemic exposure. The most successful example is N-acetylgalactosamine (GalNAc) conjugation, which targets the asialoglycoprotein receptor (ASGPR) highly expressed on hepatocytes [117,118]. Triantennary GalNAc ligands bind ASGPR with high affinity and trigger efficient internalization through clathrin-mediated endocytosis [119]. This strategy dramatically improves hepatic delivery after systemic administration, enabling dose reductions of 10–30 fold compared with unconjugated ASOs. GalNAc conjugation has now become a standard platform for liver-directed oligonucleotide drugs, with multiple approved siRNA and ASO therapeutics using this approach [117,120], substantially improved hepatocyte-specific delivery and clinical efficacy while reducing systemic exposure and dose requirements [121].

### 5.3. Lipid Conjugates

Lipid conjugation is widely explored to enhance the delivery of ASOs and siRNAs by increasing hydrophobicity and promoting interactions with membranes and circulating lipoproteins. Conjugation of lipids such as cholesterol, fatty acids (e.g., palmitic acid or C16), or other hydrophobic moieties improves tissue distribution and cellular uptake compared with unconjugated oligonucleotides [122]. These lipid-conjugated oligonucleotides often associate with plasma proteins, including low-density lipoproteins (LDL) and albumin, which facilitate transport across endothelial barriers and uptake into tissues through receptor-mediated pathways [123,124]. This strategy can enhance exposure in tissues such as liver, skeletal muscle, heart, adipose tissue, and kidney, and has been extensively applied in siRNA platforms and increasingly in antisense therapeutics [30,101].

One of the earliest and most studied lipid conjugates is cholesterol. Cholesterol-conjugated oligonucleotides bind circulating lipoproteins and are delivered to cells through lipoprotein receptors [125,126]. This mechanism promotes uptake, particularly in hepatocytes and other metabolically active tissues. In rodent studies, cholesterol-conjugated siRNAs have demonstrated efficient gene silencing in the liver and other tissues following systemic administration [125,127]. Similarly, conjugation with long-chain fatty acids such as palmitic acid or docosanoic acid (C22) enhances binding to serum albumin and promotes uptake in tissues with high fatty acid metabolism, including skeletal muscle and heart, improving gene silencing activity in these tissues [117,125].

More recently, systematic optimization has generated lipid-conjugated siRNA and ASO platforms designed for delivery to extrahepatic tissues, including CNS and peripheral neurons [126,128,129]. For example, the first C16-conjugated siRNA targeting CNS has entered clinical trial [130]. C16- or other long-chain lipid-conjugated siRNAs have also shown improved distribution to muscle and adipose tissues in nonhuman primates. While lipid conjugation improves tissue exposure and cellular uptake, variability in tissue targeting needs to be further addressed. Continued development of optimized lipid chemistries and linker strategies may further enhance the potency and tissue specificity.

### 5.4. Peptide-Mediated Delivery

Peptide-mediated delivery of oligonucleotides has been extensively developed for steric-blocking ASOs, particularly PMOs, whose neutral backbone limits cellular uptake (Table 2). The most established strategy is cell-penetrating peptide (CPP)–PMO conjugates, often referred to as PPMOs, in which arginine-enriched, cationic or amphipathic peptides are covalently linked to PMOs [131,132]. CPPs enhance binding to negatively charged cell surfaces and promote endocytosis, including macropinocytosis and caveolae-like pathways [133,134]. This conjugation enables substantially greater splice-switching activity in skeletal and cardiac muscle compared with unconjugated PMO [135]. Several optimized CPP scaffolds, including the Pip peptide series, have demonstrated marked improvements in exon-skipping efficiency and dystrophin restoration in DMD models [136,137].

Clinically, peptide–PMO conjugation (PPMO) has been pursued most prominently in DMD, where efficient delivery to skeletal and cardiac muscle is essential. A PPMO drug, SRP-5051, advanced into human trials and demonstrated improved muscle exposure compared with unconjugated PMO, yet raised safety concerns [138]. These findings illustrate a central trade-off of CPP-based delivery: while peptide conjugation substantially improves potency and tissue uptake, broad tissue exposure and strong peptide–membrane interactions may narrow the therapeutic window, particularly in the kidney and other clearance organs [14,101]. In addition, peptide-PMO conjugation has also been used to deliver oligonucleotides to other tissues such as eye and kidney (Table 2).

Recent advances have focused on improving delivery efficiency while mitigating toxicity. In particular, cyclic and bicyclic peptides have emerged as promising carriers because conformational constraint can enhance protease resistance, receptor engagement, and membrane interaction [139,140,141]. Cyclic CPPs and bicyclic peptide scaffolds have shown improved intracellular delivery of oligonucleotides and enhanced activity in preclinical models compared with linear CPPs [141]. In addition, cyclic peptides may promote more efficient endosomal escape, a major barrier to oligonucleotide activity [142,143]. Other emerging approaches include D-peptides or retro-inverso peptides to increase metabolic stability, as well as tissue-homing peptides that bind specific receptors to improve cell-type selectivity [144]. Continued optimization of peptide sequence, hydrophobic domains, linker chemistry, and formulation properties is expected to improve the balance between delivery efficiency, tissue specificity, and safety.

### 5.5. Antibody-Mediated Delivery

Antibody–oligonucleotide conjugates (AOCs) extend ASO delivery beyond “passive” uptake by leveraging receptor-mediated endocytosis (and, for CNS, receptor-mediated transcytosis). An antibody (or engineered Fc/Fab) binds a surface receptor, internalizes into endosomes, and the oligonucleotide payload must then escape to the cellular productive compartment [145]. The main advantages of AOC are cell-type selectivity (antigen-driven uptake), the ability to dose systemically (IV/SC) for tissues that are otherwise poorly reached, and the modularity to carry different oligo modalities. Key concerns include (i) endosomal escape remains rate-limiting, (ii) CMC complexity/heterogeneity (DAR/oligo loading, site of conjugation, linker stability, aggregation), and (iii) on-target/off-tissue biology of the receptor (e.g., transferrin receptor TfR1 on multiple tissues) plus potential payload-driven liabilities in clearance organs [10,146].

For muscle, anti-TfR1 AOCs have shown strong functional delivery in NHP and have advanced clinically (Table 3). In a translational study, anti-TfR1 AOCs delivered ASO/PMO modalities to striated muscle in mice [146], demonstrating robust, relatively muscle-selective pharmacology. An aTfR1 conjugated ASO can achieve ~75% mRNA knockdown in skeletal muscle at a dose 25-fold lower than an unconjugated ASO with similar knockdown. Clinically, AOC 1044 is an anti-TfR1 antibody–PMO conjugate designed to alter splicing of dystrophin. In addition, DYNE-101, a TfR-targeting Fab conjugated ASO targeting DMPK RNA, also entered clinical trials. The programs provide human feasibility for systemic, receptor-guided oligonucleotide delivery to muscle [147,148]. Recent pharmaceutical advances in AOCs have focused on optimizing linker chemistry, payload loading, manufacturability, and receptor selection, further supporting the development of next-generation AOC platforms for extrahepatic delivery of oligonucleotide therapeutics [10].

For the CNS across the blood–brain barrier (BBB), antibody-based “shuttles” targeting receptors such as TfR1 can enable transcytosis across brain endothelium, an approach now being adapted for oligonucleotides [149]. An engineered TfR1-binding “oligonucleotide transport vehicle” (OTV) has been reported to deliver a tool ASO across the BBB with broad CNS biodistribution in NHP after IV administration [150]. These efforts highlight an active design space to balance BBB transport, systemic PK, and specificity.

## 6. ASO Pharmacokinetics and Tissue Distribution

ASOs can be administered through several routes depending on the target tissue, such as subcutaneous (SC), intravenous (IV), intraperitoneal (IP), and intrathecal (IT) injection. After systemic administration, ASOs rapidly distribute from plasma to tissues, typically reaching peak plasma concentrations within 2–4 h, with plasma half-lives of several hours. Despite this short plasma exposure, ASOs exhibit extensive tissue accumulation and long intracellular persistence, largely driven by PS backbone interactions with plasma and cellular proteins [14,151].

Following systemic administration, ASOs distribute across different tissues, with the largest fraction accumulating in the liver and kidney, and significantly lower levels observed in spleen, lymph nodes, bone marrow, and other organs [3,152]. Recent quantitative analyses of tissue pharmacokinetics have further refined our understanding of tissue-specific uptake, retention, and clearance of ASOs across multiple organs, supporting more rational design of next-generation delivery strategies [153]. Within the liver, ASOs distribute to multiple cell types, including hepatocytes, Kupffer cells (liver macrophages), and sinusoidal endothelial cells. Hepatocytes generally represent the major pharmacologically relevant cells for many therapeutic targets. In the kidney, higher ASO levels were found in cortex compared with medulla. ASOs accumulate mainly in proximal tubular epithelial cells and distal tubular epithelial cells, reflecting the role of the kidney in oligonucleotide filtration and reabsorption [154,155]. In a polycystic kidney disease model, ASOs were found to be present in cyst epithelial cells [156]. Upon systemic administration, moderate levels of uptake are also observed in skeletal muscle, adipose tissue, lung, and heart [153], although delivery efficiency in these tissues is typically lower without targeting strategies.

Systemically administered ASOs can barely enter CNS due to the presence of the blood–brain barrier. However, with IT injection, PS ASOs rapidly distribute along the neuraxis via CSF flow, reaching brain regions within hours [157]. In the brain, ASOs showed broad but heterogeneous distribution, with higher exposure in cortex, spinal cord, and periventricular regions; and lower exposure in deep brain nuclei (e.g., striatum) [157]. Similarly, ASO activity was also found in different cell types, such as neurons, oligodendrocytes, astrocytes, and microglia [158].

Importantly, pharmacodynamic effects often persist far longer than plasma exposure because ASOs remain retained within intracellular compartments for days to weeks [159,160]. This prolonged tissue residence contributes to durable target knockdown and supports infrequent dosing schedules in clinical applications. [161,162]. Consequently, the endosomal escape of ASOs represents a major rate-limiting step for oligonucleotide activity, influencing both efficacy and dose requirements [3,163].

## 7. Intracellular Trafficking and Endosomal Escape

**Figure 3:**
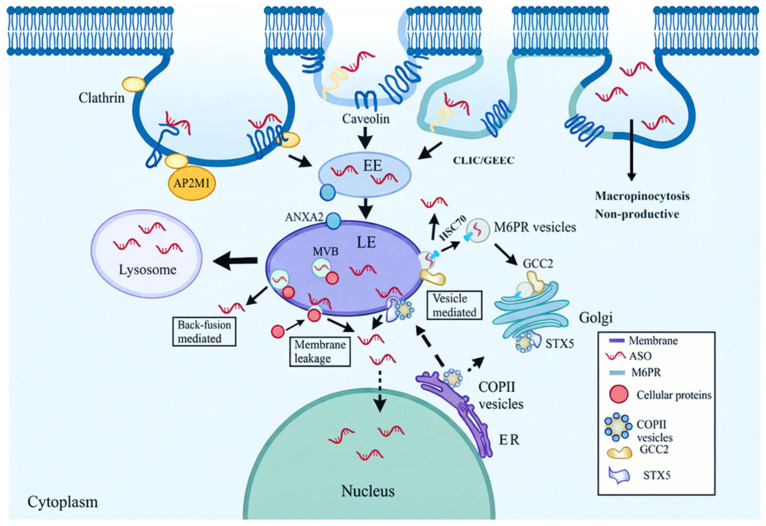
Mechanisms of PS-ASO cellular internalization and endosomal release. PS-ASOs interact with cell surface proteins that facilitate cellular uptake. They enter cells via multiple endocytic pathways, including clathrin- or caveolin-mediated micropinocytosis and macropinocytosis. Following internalization, PS-ASOs traffic from early endosomes (EE) to late endosomes (LE) and subsequently to lysosomes, likely along microtubules. Productive release predominantly occurs from late endosomes. Multiple release mechanisms may coexist, including vesicle-mediated release, back-fusion, and membrane leakage. Certain cellular proteins can relocalize to late endosomes upon PS-ASO uptake, facilitating ASO release or vesicular redirection. Once released, PS-ASOs interact with intracellular proteins and can translocate to the nucleus. MVB, multivesicular bodies; CLIC/GEEC, clathrin-independent carriers/glycosylphosphatidylinositol-anchored protein-enriched endosomal compartments.

ASOs primarily enter cells through endocytic pathways, including clathrin-mediated endocytosis, caveolin-mediated uptake, and macropinocytosis [21,164]. After internalization, ASOs traffic through the endosomal system, progressing from early endosomes (EE) to late endosomes (LE) and lysosomes. Only a small fraction of internalized ASOs escape from endosomal compartments into the cytosol or nucleus [3,163]. Despite significant progress in ASO chemistry and delivery technologies, endosomal escape remains one of the major rate-limiting barriers governing intracellular pharmacological activity and therapeutic potency of nucleic acid medicines [162]. Several cellular events have been implicated in this escape process, including endosomal maturation [165,166,167], membrane fusion [168,169], and interactions with intracellular trafficking proteins [165,167,170] (Figure 3), although the precise mechanisms governing productive release remain incompletely understood.

Despite this inefficiency, endosomal sequestration, however, may contribute to the long durability observed for oligonucleotide drugs. Studies have shown that siRNAs can be slowly released from endosomal/lysosomal reservoirs over extended periods, providing a sustained supply of active siRNAs [171]. This gradual release mechanism contributes to the long duration of gene silencing observed with siRNA therapeutics, which can persist for weeks or months in vivo after a single dose. Similar principles may apply to ASOs, where slow release from endosomes/lysosomes to the cytosol and the nucleus can sustain antisense activity. Thus, while limited endosomal escape restricts the fraction of oligonucleotide that is immediately active, the presence of intracellular reservoirs may simultaneously support prolonged target modulation and extended dosing intervals [3,30,172].

### 7.1. Cellular Factors Influencing ASO Release

Intracellular trafficking pathways play an important role in the endosomal release of ASOs. After endocytic uptake, ASOs traffic through EE, LE, and lysosomes. Within LEs, ASOs can be LE membrane associated or in multivesicular bodies (MVBs). Productive antisense activity depends on a small fraction of ASOs released from these membraned vesicles. It has been shown that endosome/lysosome movements along microtubules and perinuclear localization positively affect ASO activity [170,173,174,175]. Specific components of vesicular trafficking pathways have been found to influence this release process. For example, COPII vesicles, which normally mediate trafficking between the endoplasmic reticulum (ER) and Golgi apparatus, have been implicated in productive ASO release by relocalization to LEs after ASO uptake [176].

Other intracellular transport pathways also contribute to ASO release (Figure 3). Golgi-associated trafficking proteins, including components of the Golgi tethering (e.g., GCC2) and vesicle transport machinery (e.g., STX5), have been shown to positively regulate ASO release and activity by relocalization to LEs [165,176], indicating functional connections between the LEs and Golgi-mediated vesicle transport. In addition, the mannose-6-phosphate receptor (M6PR) pathway, which normally directs lysosomal enzyme trafficking between the Golgi and endosomes and mediates internalization of extracellular lysozymes, positively influences ASO release [165]. Furthermore, Golgi-58K, a Golgi protein that can relocate to LEs upon ASO uptake, also affects ASO activity likely by affecting GCC2 relocalization, in a Hsc70-dependent manner [170,177]. Together, these findings suggest that productive ASO release is closely linked to endosomal maturation and vesicular transport networks between ER, Golgi, and endosomes. Multiple pathways may act in parallel to contribute to ASO release, for example, protein binding to LE that may trigger membrane leakage; vesicle mediated escape; and back-fusion mediated release of ASOs present in MVBs within LEs (Figure 3). Thus, modulating these pathways may provide new strategies to enhance antisense potency.

### 7.2. ASO’s Physicochemical Properties May Affect ASO Release and Activity

Chemical modification and structural design of ASOs strongly influence their intracellular trafficking and efficiency [178]. Recent screening efforts have identified novel classes of endosomal escape enhancers capable of increasing productive intracellular delivery of oligonucleotides, providing potential combination strategies for improving ASO efficacy [161]. Backbone and sugar modifications alter hydrophobicity, charge distribution, and interactions with cellular membranes and proteins [105], which in turn affect vesicular trafficking and productive release [179]. For instance, PS backbone linkages increase interactions with membrane proteins and intracellular trafficking factors, leading to enhanced uptake and relocalization of the proteins to LEs [35,165,180]. Similarly, high-affinity sugar modifications such as 2′-MOE, cEt, and LNA can influence ASO–protein interactions and intracellular localization [48,49]. These interactions affect how ASOs partition within endosomal membranes and may modulate the probability of escape into the cytosol. Structural features such as ASO length, sequence composition, and gapmer architecture also affect trafficking behavior and antisense activity by altering binding affinity, intracellular stability, and localization [3,19,180,181].

Conjugation strategies further modulate intracellular trafficking and endosomal release by introducing chemical groups that interact with cellular membranes or receptors. Hydrophobic lipid conjugates (e.g., cholesterol or long-chain fatty acids) can enhance membrane association and promote endosomal destabilization, potentially improving cytosolic release of the ASOs [182,183]. Likewise, ligand conjugates, such as GalNAc or receptor-targeting peptides, alter the route of endocytosis and intracellular trafficking. In some cases, specific conjugates, such as CPPs, appear to favor trafficking through endosomal pathways that are more permissive for escape [184]. Despite this understanding, endosomal release remains a major barrier to oligonucleotide efficacy. Approaches have been attempted to enhance ASO release, e.g., using small molecules that boost ASO endosomal escape [178]. Enhanced ASO activity has been observed with this approach. However, it remains to be demonstrated regarding the effects on safety and duration of ASOs [101,163].

## 8. Therapeutic Applications of ASOs

Since the first ASO drug fomivirsen (Vitravene) was approved in 1998 for cytomegalovirus retinitis, the field has expanded rapidly. As of 2025, about 13 antisense oligonucleotide therapeutics have been approved (Table 4), demonstrating clinical success across different diseases [185,186]. These drugs act through diverse mechanisms, including RNase H–mediated cytoplasmic mRNA and nuclear pre-mRNA degradation and splicing modulation, highlighting the broad therapeutic flexibility of the ASO modality.

Beyond approved therapies, the ASO field continues to expand rapidly. More than 60 ASO therapeutics have entered clinical development, with over 25 programs in Phase II or III trials, targeting a broad range of indications including Huntington’s disease, Alzheimer’s disease, cancer, cardiometabolic disorders, inflammatory diseases, and viral infections [2,6,8]. Tissue targeting has also extended beyond the liver and kidney to the brain, muscle, lung, eye, and peripheral neurons, driven by advances in delivery technologies [101,187,188]. Although downregulation-based approaches remain predominant, upregulation strategies are increasingly emerging with the maturation of novel technologies.

The ASO platform has attracted substantial industrial investment, with numerous biotechnology and pharmaceutical companies actively advancing antisense programs. Collectively, these developments underscore the versatility of ASO therapeutics across diverse disease areas and biological mechanisms. Continued progress in chemical modification, targeted delivery, and the understanding of RNA biology and disease pathogenesis, together with AI-enabled target identification and drug discovery, is enabling antisense therapies to expand beyond rare diseases toward more prevalent indications, further establishing ASOs as a major modality in precision medicine.

## 9. Future Opportunities of ASO Therapeutics and Perspectives

ASO therapeutics present substantial future opportunities, because they enable bidirectional modulation of gene expression, allowing for either downregulation or upregulation of protein expression. This bidirectional control makes ASOs particularly valuable for diseases driven by either toxic gain-of-function mutations (e.g., neurodegenerative diseases such as ALS or Huntington’s disease) or haploinsufficiency disorders. In addition, increasing key pathway protein(s) that could inhibit disease progression may offer a different approach to treat chronic diseases. More broadly, recent analyses suggest that continued improvements in delivery, tissue targeting, and RNA biology will facilitate expansion of ASO therapeutics beyond rare genetic diseases into larger patient populations affected by common neurological, metabolic, cardiovascular, and inflammatory disorders [189].

Future ASO applications are expected to extend across a broad range of disease areas and tissues. Current successes have largely been achieved in the liver and CNS, where delivery strategies such as GalNAc conjugation or intrathecal administration enable efficient tissue exposure. However, growing interest is focused on expanding ASO delivery to muscle, kidney, lung, heart, and immune cells, enabling treatment of various diseases in different tissues. Advances in ligand-mediated targeting, antibody conjugates, and lipid nanoparticle formulations may enable more selective and efficient delivery to these tissues, with great opportunities for approaches that can safely and effectively traverse the blood–brain barrier and achieve efficient CNS targeting. Meanwhile, improvements in chemical modifications will continue to enhance ASO potency and stability while minimizing immune stimulation and off-target effects [30,101].

Despite these advances, several challenges remain for the next generation of ASO therapeutics. Safe and effective extrahepatic delivery of ASOs remains to be validated in the clinic. Another major area is the identification and validation of new targets involved in diseases, including regulatory ncRNAs, alternative splice isoforms, and sequence elements that control gene expression. In addition, improving intracellular delivery and endosomal escape is also critical. Achieving the optimal balance between potency, durability, and safety remains an important focus, since high-affinity chemistries and strong protein binding can influence tissue accumulation and safety, particularly in organs such as the liver and kidney. Furthermore, long-term safety, immune activation, and sequence-specific off-target hybridization must be carefully evaluated as ASO therapies move toward chronic treatment of common diseases [3,15,163].

Looking ahead, advances in RNA biology, genomics, and AI-facilitated precision medicine are expected to accelerate the development of ASO therapeutics. Integration of transcriptomics, genetic studies, and RNA structural analysis will facilitate identification of new regulatory targets, such as structures in junctions of pre-miRNAs [87], while improved delivery technologies will broaden the range of treatable tissues. In parallel, development of next-generation chemistries and targeted conjugates may further enhance therapeutic index. The emergence of patient-customized antisense oligonucleotide therapeutics has established a new paradigm for treating ultra-rare genetic diseases, exemplified by N-of-1 approaches such as Milasen and subsequent individualized ASO programs [190]. Collaborative initiatives are developing regulatory, clinical, and data-sharing frameworks to facilitate broader implementation of individualized nucleic acid medicines [191]. Together, these innovations position ASO drugs as an increasingly important modality for precise and programmable regulation of gene expression, with the potential to address both rare genetic diseases and more common complex disorders in the near future [6,101].

## Figures and Tables

**Table 1:** Comparison of Major 2′ Ribose Modifications Used in Antisense Oligonucleotides.

Modification	Structural Feature	ΔTm per Modification *	Nuclease Resistance	Typical Applications	Safety Considerations
2′-O-methyl (2′OMe)	Methyl substitution at 2′-OH	~0.5–1 °C	Moderate	Early ASO, siRNA stabilization	Generally well tolerated
2′-O-methoxyethyl (MOE)	Methoxyethyl substituent	~0.5-1 °C	High	Clinical ASO drugs, gapmer wings	Favorable safety profile
Constrained ethyl (cEt)	Bicyclic ribose constraint	~2–5 °C	Very high	Next-generation gapmers	Requires sequence optimization
Locked nucleic acid (LNA)	2′-O–4′-C methylene bridge	~2–5 °C	very high	High-affinity ASOs, miRNA inhibition	Requires optimization

* Approximate increase in duplex melting temperature relative to DNA.

**Table 2:** Peptide mediated delivery of oligonucleotides in clinical studies.

Compound	Sponsor	ASO Chemistry	Peptide Delivery	Molecular Target	Target Tissue	Route
SRP-5051	Sarepta Therapeutics	PMO exon-skipping ASO	Cell-penetrating peptide	dystrophin pre-mRNA	Muscle	intravenous
VP-001	PYC Therapeutics	PMO splice-modulating ASO	Cell-penetrating peptide–	CNOT3 pre-mRNA	Eye	Intravitreal
PYC-001	PYC Therapeutics	PMO Translation enhancing	Cell-penetrating peptide	OPA1 mRNA	Eye	Intravitreal
PYC-003	PYC Therapeutics	PMO, miRNA blocking	Peptide	PKD1 mRNA	Kidney	intravenous

**Table 3:** Antibody conjugation of oligonucleotides in clinical trials.

Conjugate/ASO	Antibody Type	Target Tissue/Cells	Species Tested
αTfR1 AOC platform: ASO/siRNA/PMO payloads	anti-TfR1 monoclonal antibody	Skeletal muscle, heart	mouse, cynomolgus monkey
DYNE-101, DMPK ASO	anti-TfR1 Fab, Val-Cit cleavable linker	Skeletal muscle; DM1-relevant muscle cells	mouse models, cynomolgus monkey
AOC 1044, PMO44	anti-TfR1 monoclonal antibody	Skeletal and cardiac muscle; DMD exon 44 skipping	DMD mouse model, cynomolgus monkey
AOC 1020, siDUX4.6	anti-TfR1 mAb AV01mAb	Skeletal muscle; FSHD/DUX4	FSHD mouse model, cynomolgus monkey

**Table 4:** Approved Antisense Oligonucleotide (ASO) Drugs.

Drug (Brand)	Target	Mechanism of Action	Company/Developer	Approval Year	Conjugate	Core Chemistry
Fomivirsen (Vitravene)	CMV IE2 mRNA	RNaseH degradation	Isis (Ionis)/Novartis	1998	None	PS-DNA
Mipomersen (Kynamro)	ApoB mRNA	RNase H degradation	Ionis/Sanofi	2013	None	PS gapmer (2′-MOE wings)
Nusinersen (Spinraza)	SMN2 pre-mRNA	Splice modulation (exon 7 inclusion)	Ionis/Biogen	2016	None	PS 2′-MOE
Eteplirsen (Exondys 51)	Dystrophin pre-mRNA	Exon skipping	Sarepta	2016	None	PMO
Inotersen (Tegsedi)	TTR mRNA	RNase H degradation	Ionis	2018	None	PS gapmer (2′-MOE)
Volanesorsen (Waylivra)	APOC3 mRNA	RNase H degradation	Ionis	2019 (EU)	None	PS gapmer
Golodirsen (Vyondys 53)	Dystrophin pre-mRNA	Exon skipping	Sarepta	2019	None	PMO
Viltolarsen (Viltepso)	Dystrophin pre-mRNA	Exon skipping	NS Pharma/Nippon Shinyaku	2020	None	PMO
Casimersen (Amondys 45)	Dystrophin pre-mRNA	Exon skipping	Sarepta	2021	None	PMO
Tofersen (Qalsody)	SOD1 mRNA	RNase H degradation	Ionis/Biogen	2023	None	PS gapmer (2′-MOE)
Eplontersen (Wainua)	TTR mRNA	RNase H degradation	Ionis/AstraZeneca	2023	GalNAc	PS gapmer (2′-MOE)
Olezarsen (Tryngolza)	APOC3 mRNA	RNase H degradation	Ionis	2024	GalNAc	PS gapmer (2′-MOE)
Donidalorsen	KLKB1 mRNA	RNase H degradation	Ionis	2025	GalNAc	PS gapmer (2′-MOE)

## Data Availability

No new data were created or analyzed in this study. Data sharing is not applicable to this article.

## Abbreviations

The following abbreviations are used in this manuscript

ALS	Amyotrophic lateral sclerosis
ANA	Arabinonucleic acid
AOC	Antibody–oligonucleotide conjugates
ASO	Antisense Oligonucleotides
cEt	Constrained ethyl
DMD	Duchenne muscular dystrophy
GalNAc	N-acetylgalactosamine
GNA	Glycol nucleic acid
LNA	Locked Nucleic Acid
LNP	Lipid nanoparticles
me	2′-O-methyl
MOE	2′-O-Methoxyethyl
NMD	Nonsense mediated decay
PMO	Phosphorodiamidate morpholino oligomer
PNA	Peptide Nucleic Acid
PO	Phosphodiester
PS	Phosphorothioate
PTC	Premature stop codon
SMA	Spinal muscular atrophy
TIE	Translation inhibitory element
UNA	Unlocked nucleic acid
